# *CFH-CFHR1* hybrid genes in two cases of atypical hemolytic uremic syndrome

**DOI:** 10.1038/s10038-023-01129-1

**Published:** 2023-02-09

**Authors:** Yuka Sugawara, Hideki Kato, Masao Nagasaki, Yoko Yoshida, Madoka Fujisawa, Naoko Minegishi, Masayuki Yamamoto, Masaomi Nangaku

**Affiliations:** 1 Division of Nephrology and Endocrinology, The University of Tokyo Graduate School of Medicine, Tokyo, Japan; 2 Center for Genomic Medicine, Graduate School of Medicine, Kyoto University, Kyoto, Japan; 3 Tohoku Medical Megabank Organization, Tohoku University, Miyagi, Japan

**Keywords:** Disease genetics, Immunological disorders

## Abstract

Atypical hemolytic uremic syndrome (aHUS) is a rare complement-mediated disease that manifests as the triad of thrombotic microangiopathy. We identified two aHUS patients with neither anti-complement factor H (CFH) antibodies nor causative variants of seven aHUS-related genes (*CFH*, *CFI*, *CFB*, *C3*, *MCP*, *THBD*, and *DGKE*); however, their plasma showed increased levels of hemolysis by hemolytic assay, which strongly suggests CFH-related abnormalities. Using a copy number variation (CNV) analysis of the *CFH/CFHR* gene cluster, we identified *CFH-CFHR1* hybrid genes in these patients. We verified the absence of aHUS-related abnormal CNVs of the *CFH* gene in control genomes of 2036 individuals in the general population, which suggests that pathogenicity is related to these hybrid genes. Our study emphasizes that, for patients suspected of having aHUS, it is important to perform an integrated analysis based on a clinical examination, functional analysis, and detailed genetic investigation.

## Background and case reports

Atypical hemolytic uremic syndrome (aHUS) is a rare disease resulting from the dysregulation of the alternative complement pathway and manifests as the triad of thrombotic microangiopathy (TMA) [1]: microangiopathic hemolytic anemia, thrombocytopenia, and acute kidney failure [2, 3].

Genetic abnormalities in some complement or coagulation-related genes, such as *CFH*, *CFB*, *CFI*, *C3*, *MCP*, *THBD*, and *DGKE*, or acquired autoantibodies against complement factor H (CFH) were reported to cause aHUS; however, these abnormalities occurred in only 58% to 70% of patients with aHUS, and thus could not fully explain causality [4].

CFH is the main regulator of the alternative complement pathway and is composed of 20 short consensus repeats (SCRs). *CFH* and its five downstream genes, *CFHR3*, *CFHR1*, *CFHR4*, *CFHR2*, and *CFHR5*, which encode for complement factor H-related (CFHR) proteins, comprise the *CFH/CFHR* gene cluster at chromosome 1q32. This cluster contains multiple highly homologous sequences, likely generated by gene duplication or deletion events [5–7]. Several types of hybrid genes have been reported in the *CFH/CFHR* gene cluster of aHUS patients [8–13]. This brief communication reports two cases of Japanese aHUS patients with *CFH-CFHR1* hybrid genes, carrying novel different breakpoints, and this is the first time that hybrid genes are reported as a cause of aHUS in Japanese cases.

**Fig. 1:**
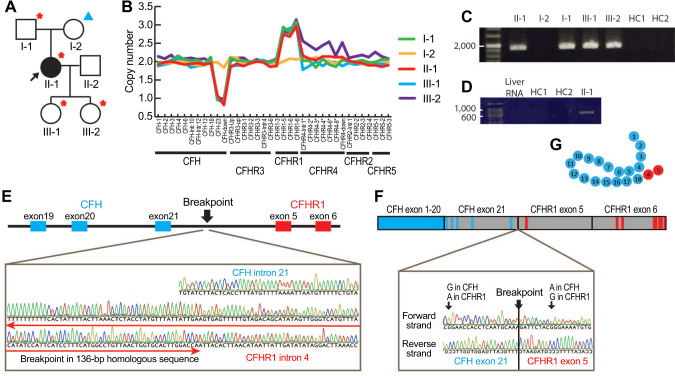
*CFH-CFHR1* hybrid gene in patient-1. **A** Family tree. The affected case (II-1) is represented by a solid-black circle. Family members with and without the *CFH-CFHR1* hybrid gene are indicated with a red asterisk and blue triangle, respectively. II-2 was not analyzed. **B** Copy number determination of the *CFH/CFHR* gene cluster using the multiplex ligation-dependent probe analysis identified a heterozygous deletion of *CFH* exon 23 to its downstream sequence and heterozygous duplication of *CFHR1* exons 5 to 6. Probes generated in-house are indicated with asterisks. **C** The genomic breakpoint was confirmed by long PCR amplification with break-point specific primers. Bands are shown with 200-bp DNA ladder (Takara Bio. Inc.). The predicted size of the bands was 1925 bp. HC, healthy control. **D** A mRNA for the *CFH-CFHR1* hybrid gene was confirmed by amplifying the patient cDNA with hybrid-specific primers. Bands are shown with 50 bp DNA ladder (Takara Bio. Inc.). The predicted size of the band was 818 bp. Total human liver RNA is also used as a control, because complement proteins are mainly produced in liver. HC healthy control. **E** Schematic presentation of the *CFH-CFHR1* hybrid gene and chromatogram showing the genomic breakpoint. **F** Schematic presentation of the mRNA for the *CFH-CFHR1* hybrid gene and chromatogram demonstrating its breakpoint. Blue and red lines represent *CFH*-specific and *CFHR1*-specific single nucleotide polymorphisms, respectively. The gray area represents the homologous sequence between *CFH* and *CFHR1*. **G** Putative structure of the CFH-CFHR1 hybrid protein. Blue and red circles denote SCRs that originated from CFH and CFHR1, respectively

**Fig. 2:**
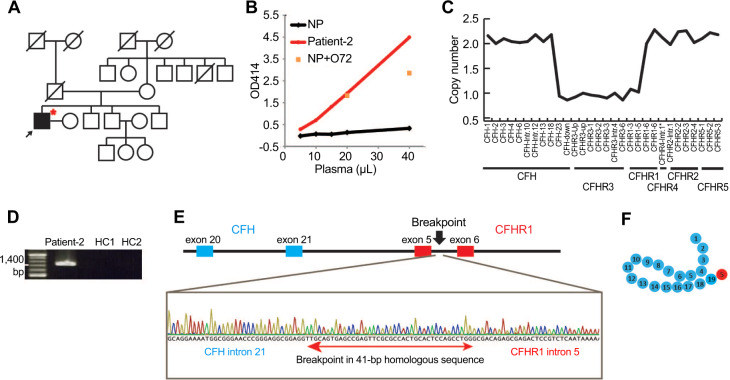
*CFH-CFHR1* hybrid gene in patient-2. **A** Family tree. The affected case is represented by a solid-black square. A red asterisk indicates the presence of the *CFH-CFHR1* hybrid gene. **B** Hemolytic assay results. The plasma sample of patient-2 induced strong hemolysis of sheep red blood cells. NP normal plasma. NP + O72, normal plasma spiked with anti-CFH mAb O72. **C** The multiplex ligation-dependent probe analysis revealed a heterozygous deletion of *CFH* exon 23 to *CFHR1* exon 5. Probes generated in-house are indicated with asterisks. **D** The genomic breakpoint was confirmed by long PCR amplification with break-point specific primers. Bands are shown with 200 bp DNA ladder (Takara Bio. Inc.). The predicted size of the band was 1483 bp. HC healthy control. **E** Schematic presentation of the *CFH-CFHR1* hybrid gene and chromatogram showing the genomic breakpoint within a 41-bp homologous sequence. Yellow circles highlight G bases, green circles highlight T bases, and blue circles highlight C bases, indicating differences before and after the homologous site where recombination occurred. **F** Putative structure of the CFH-CFHR1 hybrid protein. Blue and red circles denote SCRs that originated from CFH and CFHR1, respectively

This study was approved by the ethics committee of The University of Tokyo Hospital (IRB G10029) and registered to the UMIN-CTR (UMIN000014869). Written informed consent was obtained from all participants. The case of patient-1 (II-1, Fig. 1A), a 36-year-old female, was previously reported [14]. The occurrence of a common genetic abnormality in the plasma of patient-1 and members of her family (I-1, III-1, and III-3) was deduced from a hemolytic assay using sheep red blood cells [14] (a functional assay to detect complement dysregulation mainly associated to CFH). Patient-2 (Fig. 2A) was a 55-year-old male who presented with TMA and was successfully treated by plasma exchange. Similar to patient-1, the hemolytic assay for patient-2 also showed increased hemolysis indicating CFH-related abnormalities (Fig. 2B). However, a screening of aHUS-related genes (*CFH*, *CFI*, *CFB*, *C3*, *MCP*, *THBD*, and *DGKE*) did not identify any pathogenic variants, and autoantibodies against CFH were absent.

Two unusual copy number variations (CNVs) were observed in the *CFH/CFHR* gene cluster using a multiplex ligation-dependent probe analysis. In patient-1 and her family members, we observed a heterozygous deletion extending from *CFH* exon 23 to its downstream sequence and a heterozygous duplication extending from *CFHR1* exon 5 to 6 (Fig. 1B). In patient-2, an unusual heterozygous deletion was observed extending from *CFH* exon 23 to *CFHR1* exon 5 (Fig. 2C).

Regarding the family of patient-1, long-range PCR confirmed the existence of a *CFH-CFHR1* hybrid gene (Fig. 1C) and revealed a breakpoint within a 136-bp region, which was homologous between *CFH* intron 21 and *CFHR1* intron 4 (Fig. 1E). cDNA sequencing for patient-1 confirmed the presence of mRNA transcripted from the *CFH-CFHR1* hybrid gene (Fig. 1D). The hybrid gene was in-frame and composed of 23 coding exons, the first 21 of which were derived from *CFH* exons 1–21 and terminal 2 from *CFHR1* exons 5–6 (Fig. 1F). The protein product is expected to be an in-frame 20-SCR protein where SCRs 1 to 18 are derived from CFH and SCR 19 to 20 from CFHR1 (Fig. 1G). Regarding patient-2, a *CFH-CFHR1* hybrid gene was also predicted to be in-frame based on the genomic sequence, and its breakpoint was within a 41-bp region, which was homologous between *CFH* intron 22 and *CFHR1* intron 5 (Figs. 2D, E). The length of the deletion was 84.6 kb, extending from *CFH* intron 22 to *CFHR1* intron 5.

Since all the aHUS-related hybrid genes showed an abnormal copy number for the *CFH* gene C-terminus, we evaluated the copy number of the *CFH/CFHR* gene cluster in control genomes of 2036 individuals in the general population from the Tohoku Medical Megabank Project [15], using whole-genome sequencing data (described in copy number analysis section in Supplementary Notes). Although some types of CNVs were detected for *CFHR1* to *CFHR5*, the copy number of *CFH* was normal in all individuals (Supplementary Fig. 1; Supplementary Table 1). Whole genome analysis was also performed on the two aHUS cases reported here, and the copy number changes shown by MLPA were detected by whole genome analysis as well (Supplementary Fig. 2).

**Fig. 3:**
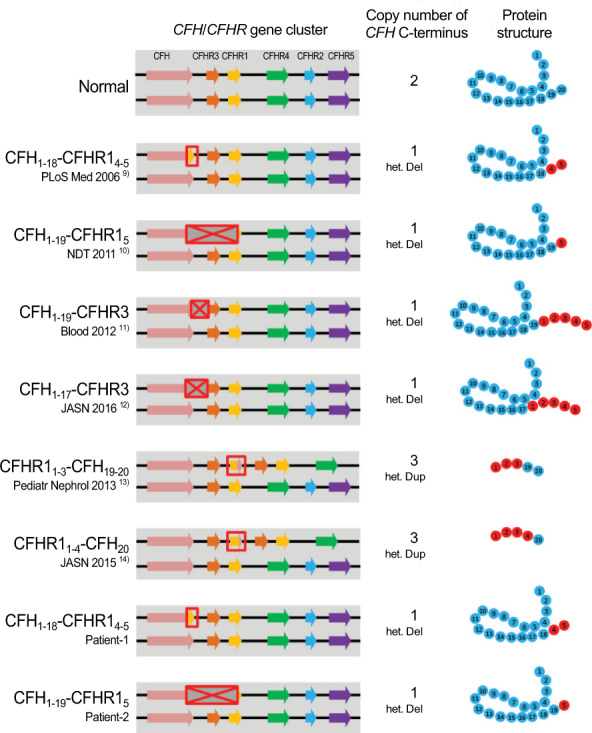
aHUS-related hybrid genes in the *CFH/CFHR* gene cluster. Previously reported aHUS cases (top six) and cases reported in this study (bottom two) with hybrid genes in the *CFH/CFHR* gene cluster. Putative structures of hybrid proteins are shown on the right. The CFH protein reportedly adopts a hinge-like arrangement in circulation. het. Del, heterozygous deletion; het. Dup, heterozygous duplication

To date, six types of hybrid genes were reported in the *CFH/CFHR* gene cluster (Fig. 3) [8–13]. In four of these, C-terminal-deficient CFH proteins were fused to the C-terminus of the CFHR protein (“CFH-CFHR protein”) [8–11], similar to our cases. Because the impaired function of the CFH C-terminus, i.e., the SCR 19–20 surface binding domains [16], results in reduced recognition of and binding to host cell surfaces, the CFH-CFHR hybrid proteins devoid of this domain can cause over-activation of the complement system on cell surfaces. On the other hand, the other two hybrid genes consisted of a C-terminal-deficient CFHR protein and C-terminus of the CFH protein (“CFHR-CFH protein”) [12, 13]. Since the CFHR-CFH proteins can bind to cell surfaces via the C-terminus of the CFH protein, they will compete with and reduce the binding of normal CFH proteins.

Notably, the *CFH-CFHR1* hybrid gene found in the family of patient-1 exhibited a reduced penetrance, which is consistent with past reports [17]. Penetrance rates of aHUS patients with the *CFH* mutation was reported to be 59% [18], and those with the *CFH-CFHR* hybrid gene showed rates of 33% [10]. The family members of patient-2 denied consent and could not be analyzed in this study. It is possible that his is a sporadic case caused by a de novo genomic rearrangement [9, 11].

In considering the pathogenicity of ultra-rare diseases, including aHUS, the frequency of each variation is expected to have a crucial role. Since no abnormal CNVs of the *CFH* gene occurred in 2 036 healthy racially-matched individuals, the variants described in this study demonstrate moderate evidence of pathogenicity (PM2) according to the American College of Medical Genetics [19]. In addition, the same type of hybrid protein has already been reported to be pathogenic in other aHUS cases [8, 9], providing strong evidence of pathogenicity (PS3). The hybrid genes presented here are classified as “likely pathogenic” due to combination of PS3 and PM2.

This study also had a limitation. We only demonstrated the breakpoints that make up the hybrid genes and did not show the breakpoints within introns. Sequences between *CFH-CFHR3* and sequences between *CFHR1-CFHR4* are highly homologous over a 30-kbp stretch [7]. Considering this, and the fact that the copy number of *CFHR3* was shown to be normal by MLPA, we speculate that the 3’ side of *CFH* was replaced by the 3’ side of *CFHR1*; however, as the sequence was restored within the highly homologous intron portion, the copy number of *CFHR3* was normal, although we were not able to show this because of the difficulty in designing appropriate primers.

In summary, we detected novel breakpoints that cause *CFH-CFHR1* hybrid genes in two aHUS cases through CNV and functional analyses, and also confirmed the absence of CNVs of the *CFH* gene in the general population. Our study emphasizes that the combination of functional and genetic analyses can more accurately elucidate the genetic origin and, ultimately, the biomolecular mechanisms of aHUS.

## Supplementary information

Supplementary Notes. Materials and methods.

Supplementary Fig. 1. Discretized copy number of the *CFH/CFHR* gene cluster in 2 036 general population

Supplementary Fig. 2. Copy number of the *CFH/CFHR* gene cluster in 2 036 individuals in the general population and two aHUS cases with hybrid genes

Supplementary Table 1. Summary of copy number variations (CNVs) in the *CFH/CFHR* gene cluster detected in 2 036 general population

ICMJE Disclosure form

Supplementary Information
