# Supplementary material for: CFH-CFHR1 hybrid genes in two cases of atypical hemolytic uremic syndrome
Source: J Hum Genet. 2023 Feb 9;68(6):427–30. doi: 10.1038/s10038-023-01129-1 (PMC10208952; doi:10.1038/s10038-023-01129-1)
Supplement: Supplementary file 1 — Supplementary Notes. Materials and methods. [file 10038_2023_1129_MOESM1_ESM.docx]

**Materials and Methods**

**Subjects**

The study was approved by the ethical committee of The University of Tokyo Hospital (IRB G10029) and registered to UMIN-CTR (UMIN000014869). Informed consent was obtained in accordance with the Declaration of Helsinki.

**Patients’ details**

Patients were found in the aHUS cohort of Japan, established in Nara Medical University and the University of Tokyo Hospital from 2014 to 2017 ^1^.

Patient-1 was a 32-year-old woman (index case, II-1, Figure 1) who presented with acute kidney failure after an episode of acute gastroenteritis. Her kidney biopsy showed crescentic nephritis and TMA. Renal function deteriorated and she required hemodialysis thereafter. She received a living-related kidney transplant from her mother at the age of 36; however, she presented a classical feature of TMA on postoperative day (POD) 2. Though none of plasma exchange, rituximab, intravenous immunoglobulin and steroid pulse therapy were effective, eculizumab started on POD 36 improved her kidney function markedly and no more TMA attack was observed for seven years thereafter. The levels of complement parameters C3 and C4 were not decreased. Anti-nuclear antibody and anti-dsDNA antibody were negative.

Patient-2 was a 55-year-old man presented with a classical feature of TMA that manifested one week after an episode of general fatigue, diarrhea and vomiting. On admission, the level of complement parameter C3 was slightly low (67 mg/dL, 80 – 140 mg/dL). After the treatment of plasma exchange and hemodialysis, his platelet counts, and hemoglobin level recovered gradually. Anti-nuclear antibody and anti-dsDNA antibody were negative. In his family, there is no other TMA or kidney disease-affected person.

**The hemolytic assay using sheep blood cells and anti-CFH antibodies screening**

The hemolytic assay using sheep red blood cells was performed as previously described ^2^. Shortly, resuspended sheep red blood cells (Japan Lamb, Japan) were incubated with a dilution series of a patient plasma sample at 37 ℃ for 30 min, and the level of hemoglobin released was measured by the absorbance at 414 nm. The hemolysis obtained from normal plasma spiked with monoclonal antibody against CFH (O72, 200μg IgG/mL, final) was defined as a 100% hemolysis as the control.

The presence of anti-CFH antibodies in each patient’s plasma were evaluated using CFH IgG ELISA kits (Abnova, KA1477) according to the manufacturer’s instructions.

**Whole-exome sequencing**

Genomic DNA from the patients, family members and healthy control individuals was extracted from peripheral blood using QIAamp DNA midi kit (QIAGEN).

The index patients were analyzed by whole-exome sequencing using HiSeq 2000 (Illumina). Sequences were aligned to the human reference genome (NCBI37/Hg19) using the Burrows-Wheeler Aligner (0.7.12-r1039) ^3^. Variant calling was performed using gatk Genome Analysis Tool kit (v3.4-46-gbc02625) ^4^ and was annotated by ANNOVAR ^5^. Mutations and polymorphisms in complement genes associated with aHUS (*CFH, CFI, CFB, C3, MCP, THBD* and *DGKE*) were screened.

**Multiplex ligation-dependent probe analysis**

Copy numbers of *CFH* and genes encoding factor H–related proteins, *CFHR3, CFHR1, CFHR2* and *CFHR5*, were analyzed by multiplex ligation-dependent probe analysis; MLPA, which was performed with the SALSA MLPA P236-A1 ARMD Kit (MRC-Holland). Additional homemade MLPA probes for *CFHR4* were also used (probe sequences available on request). Data of MLPA were analyzed by Coffalyser (MRC-Holland). Note that not all *CFH*-related copy number abnormalities can be found in MLPA, because the *CFH/CFHR* gene cluster has numerous homologous regions.

**Break-point analysis**

Based on the past reports about hybrid genes in aHUS patients ^6-11^ and our results of MLPA, we estimated chromosomal breakpoints in Patient-1 and Patient-2, respectively. Amplicons that targeted these breakpoints were obtained by PCR using KOD Fx Neo (Toyobo) and primers designed by the Primer-BLAST software (https://www.ncbi.nlm.nih.gov/tools/primer-blast/). Primer sequences used for break-point analysis are as follows:

|  | Forward | Reverse |
| --- | --- | --- |
| Patient-1 | ATAGTCTATTCACTACACATGGATA  (chr1: 196711891-196711915) | GCATGCAGAGAAAATACGAC  (chr1: 196798410-196798429) |
| Patient-2 | CTGTGATGAGTCTGATATTTCACTG  TTTG (chr1: 196715166-196715194) | AGTCCCCTACTATTACTGCATTG  TTTA (chr1: 196803149-196803175) |

Sequencing of the PCR products was performed by Macrogen company.

**cDNA sequencing**

RNA of the patients and healthy control individuals was isolated from peripheral blood using Nucleospin RNA blood (Macherey-Nagel). Human Liver RNA was purchased (Agilent Technologies, MVP total RNA, Human Liver, #540017). cDNA was synthesized using PrimeSceipt RT Master Mix (Takara Bio). We performed PCR using primers to amplify the sequence around breakpoints on cDNA. Primer sequences used for break-point analysis on cDNA are as follows:

|  | Forward | Reverse |
| --- | --- | --- |
| Patient-1 | AACAGATTGTCTCAGTTTACCTAGC (chr1:196711006-196711030, NM_000186.4, c.3033-3057) | CAGCTGATTCACCTGTTCTCA  (chr1:196801005-196801025, NM_002113, c. 983-1003) |

Sequencing of the PCR products was performed by Macrogen company.

**Copy number analysis of *CFH* gene regions in 2,036 general population.**

Tohoku Medical Megabank (TMM) Project combines population genomics, medical genetics and prospective cohort studies in the two prefectures in Japan (Miyagi and Iwate). Short-read whole-genome sequence with high coverage (32x) of 2,036 participants in TMM Project, completed in 2016, was analyzed to investigate the frequencies of copy number variation in *CFH/CFHR* gene cluster in Japanese general population. The alignment step for the 2,036 whole-genome sequenced data was the same operation in the previous paper ^12^. For the two aHUS patients whole-genome sequencing fastq files, the same alignments steps were applied (named Parient-1 and Parient-2). Notably, the whole-genome sequencing protocol was a PCR-free protocol and suitable for the downstream copy number analysis. For the 2,038 aligned bam files, a normalized read-depth coverage analysis was performed to the *CFH* to *CFHR5* gene regions, i.e. chr1:196,600,000-19,700,000 of the GRCh37 coordinate, across 500 bp sliding windows in 100 bp increments. For the normalization, global normalization and local normalization were applied. The global normalization operation rescaled the average coverage to be two copies using the total number of reads mapped to autosomes (chromosomes 1 to 22). For the local normalization, two divided by the mean value of total 2,038 samples in the same window was multiplied to the value of former global normalized step. Finally, in total the matrix of 4,000 windows and 2,038 samples were created.

For the windows corresponds to the genebody regions of *CFH* (chr1:196,620,800-196,716,300), *CFHR3* (chr1:196,743,700-196,762,900), *CFHR4* (chr1:196,819,200-196,887,800), *CFHR2* (chr1:196,912,700-196,928,100) and *CFHR5* (chr1:196,946,500-196,978,500), the mean values were calculated to estimate these copy counts. For the discretization of each value, a value less than 0.25 was treated as 0 copy, 0.75 to 1.5 as one copy, 1.75 to 2.5 as two copies and more than 2.75 as three copies. All 2,036 general control samples were successfully categorized into four groups, i.e. all estimated value were not ambiguous values between 0.25 to 0.75, 1.5 to 1.75 and 2.5 to 2.75. Instead, the normalized value of *CFH* region for two aHUS patient samples were categorized into ambiguous values 1.675 and 1.736. For the discretized gene copy counts to 2,036 general control samples, the R (version 3.6.2) package pheatmap ^13^ was used to visualize the clustered heatmap (Supplementary Fig. 1). The clustering operation for the vertical axis was the default setting of the pheatmap library (version 1.0.12). Supplementary Table 1 summarized the estimated copy counts.

To further analyze the detail of genebody regions of *CFH*, *CFHR3, CFHR1, CFHR4*, *CFHR2*, and *CFHR5,* we have applied the clustering analysis based on the normalized depth in the windows that overlap to the genebody regions of *CFH*, *CFHR3*, *CFHR1*, *CFHR4*, *CFHR2*, and *CFHR5*, and other values in the matrix were set to the same value, 2 (intergenic region). Using this operation, the matrix of 2,038 samples based on 4,000 windows has the normalized value only in gene body regions. The R (version 3.6.2) package pheatmap ^13^ was used to visualize the clustered heatmap (Supplementary Fig. 2). The clustering operation for the vertical axis was the default setting of the pheatmap library (version 1.0.12). The labels in the right indicate the source of samples, 2036 individuals in the general population in Japan, or Patient-1 and Patient-2. The two patients were clustered together in Supplementary Fig. 2. The WGS copy number status is consistent with the MLPA. In Patient-1, a heterozygous deletion extends from *CFH* exon 23 to its downstream sequence and heterozygous duplication extending from *CFHR1* exon 5 to 6. In Patient-2, an unusual heterozygous deletion extends from *CFH* exon 23 to *CFHR1* exon 5.

Reference

1. Fujisawa M, Kato H, Yoshida Y, Usui T, Takata M, Fujimoto M, et al. Clinical characteristics and genetic backgrounds of Japanese patients with atypical hemolytic uremic syndrome. Clin Exp Nephrol. 2018;22:1088-1099.
2. Yoshida Y, Miyata T, Matsumoto M, Shirotani-Ikejima H, Uchida Y, Ohyama Y, et al. A novel quantitative hemolytic assay coupled with restriction fragment length polymorphisms analysis enabled early diagnosis of atypical hemolytic uremic syndrome and identified unique predisposing mutations in Japan. PLoS One. 2015;10:e0124655.
3. Li H, Durbin R. Fast and accurate short read alignment with Burrows-Wheeler transform. Bioinformatics. 2009;25:1754-60.
4. McKenna A, Hanna M, Banks E, Sivachenko A, Cibulskis K, Kernytsky A, et al. The Genome Analysis Toolkit: a MapReduce framework for analyzing next-generation DNA sequencing data. Genome Res. 2010;20:1297-303.
5. Wang K, Li M, Hakonarso H. ANNOVAR: functional annotation of genetic variants from high-throughput sequencing data. Nucleic Acids Res. 2010;38:e164.
6. Venables JP, Strain L, Routledge D, Bourn D, Powell HM, Warwicker P, et al. Atypical haemolytic uraemic syndrome associated with a hybrid complement gene. PLoS Med. 2006;3:e431.
7. Maga TK, Meyer NC, Belsha C, Nishimura CJ, Zhang Y, Smith RJ. A novel deletion in the RCA gene cluster causes atypical hemolytic uremic syndrome. Nephrol Dial Transplant. 2011;26:739-41.
8. Francis NJ, McNicholas B, Awan A, Waldron M, Reddan D, Sadlier D, et al. A novel hybrid CFH/CFHR3 gene generated by a microhomology-mediated deletion in familial atypical hemolytic uremic syndrome. Blood. 2012;119:591-601.
9. Challis RC, Araujo GS, Wong EK, Anderson HE, Awan A, Dorman AM, et al. A De Novo Deletion in the Regulators of Complement Activation Cluster Producing a Hybrid Complement Factor H/Complement Factor H-Related 3 Gene in Atypical Hemolytic Uremic Syndrome. J Am Soc Nephrol. 2016;27:1617-24.
10. Eyler SJ, Meyer NC, Zhang Y, Xiao X, Nester CM, Smith RJ. A novel hybrid CFHR1/CFH gene causes atypical hemolytic uremic syndrome. Pediatr Nephrol. 2013;28:2221-5.
11. Valoti E, Alberti M, Tortajada A, Garcia-Fernandez J, Gastoldi S, Besso L, et al. A novel atypical hemolytic uremic syndrome-associated hybrid CFHR1/CFH gene encoding a fusion protein that antagonizes factor H-dependent complement regulation. J Am Soc Nephrol. 2015;26:209-19.
12. Nagasaki M, Yasuda J, Katsuoka F, Nariai N, Kojima K, Kawai Y, et al. Rare variant discovery by deep whole-genome sequencing of 1,070 Japanese individuals. Nat Commun. 2015;6:8018.
13. <https://www.rdocumentation.org/packages/pheatmap/versions/1.0.12/topics/pheatmap>
