# Supplementary figures and images for: CFH-CFHR1 hybrid genes in two cases of atypical hemolytic uremic syndrome
Source: J Hum Genet. 2023 Feb 9;68(6):427–30. doi: 10.1038/s10038-023-01129-1 (PMC10208952; doi:10.1038/s10038-023-01129-1)

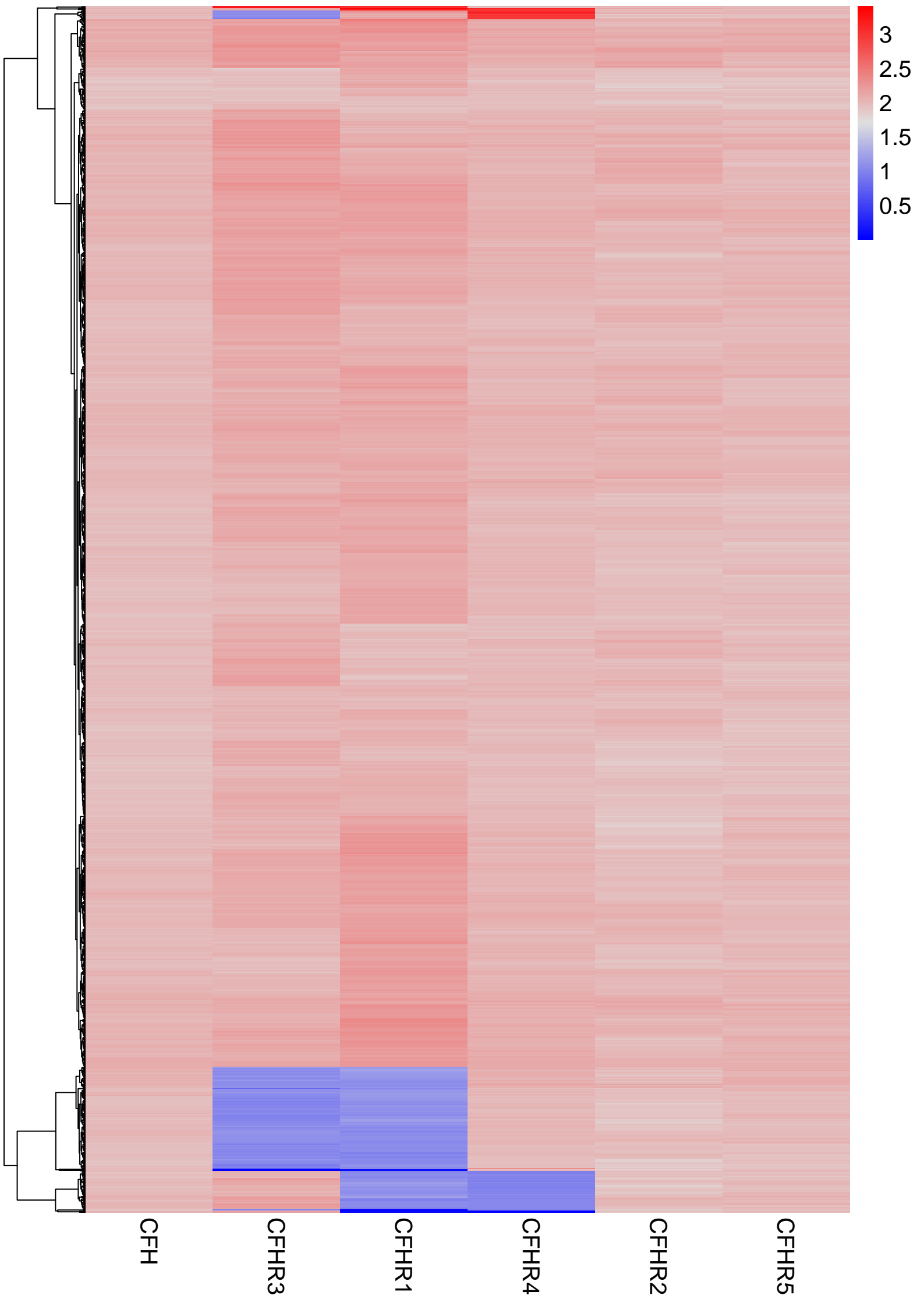

Supplement: Supplementary file 2 — Supplementary Fig. 1. Discretized copy number of the CFH/CFHR gene cluster in 2 036 general population [file 10038_2023_1129_MOESM2_ESM.pdf]

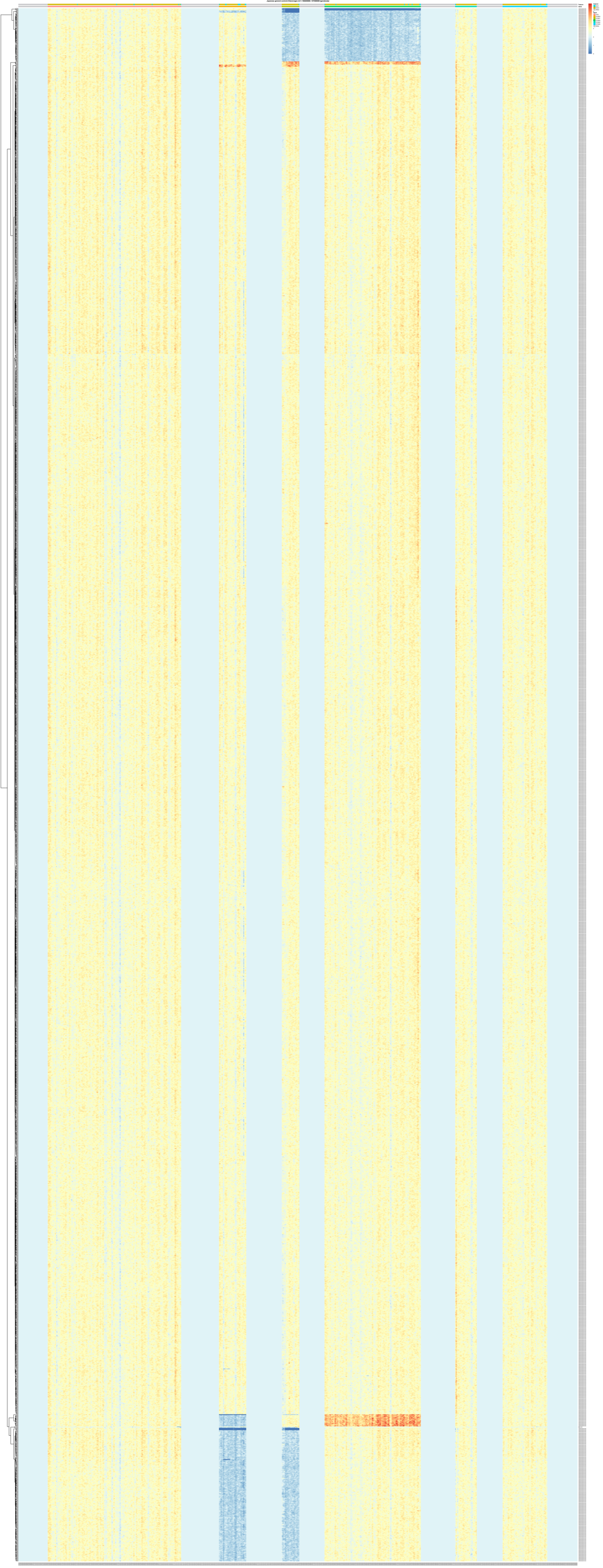

Supplement: Supplementary file 3 — Supplementary Fig. 2. Copy number of the CFH/CFHR gene cluster in 2 036 individuals in the general population and two aHUS cases with hybrid genes [file 10038_2023_1129_MOESM3_ESM.pdf]
