# Supplementary material for: CFH-CFHR1 hybrid genes in two cases of atypical hemolytic uremic syndrome
Source: J Hum Genet. 2023 Feb 9;68(6):427–30. doi: 10.1038/s10038-023-01129-1 (PMC10208952; doi:10.1038/s10038-023-01129-1)
Supplement: Supplementary file 4 — Supplementary Table 1. Summary of copy number variations (CNVs) in the CFH/CFHR gene cluster detected in 2 036 general population [file 10038_2023_1129_MOESM4_ESM.docx]

| Pattern | N (%) | Copy number | | | | | | Comments |
| --- | --- | --- | --- | --- | --- | --- | --- | --- |
|  |  | *CFH* | *CFHR3* | *CFHR1* | *CFHR4* | *CFHR2* | *CFHR5* |  |
| A | 3 (0.15%) | **2** | 0▼ | 0▼ | 2 | 2 | 2 | Homozygous *CFHR3/CFHR1* deletion |
| B | 172 (8.45%) | **2** | 1▼ | 1▼ | 2 | 2 | 2 | Heterozygous *CFHR3/CFHR1* deletion |
| C | 3 (0.15%) | **2** | 3▲ | 3▲ | 2 | 2 | 2 | Heterozygous *CFHR3/CFHR1* duplication |
| D | 3 (0.15%) | **2** | 2 | 0▼ | 0▼ | 2 | 2 | Homozygous *CFHR1/CFHR4* deletion |
| E | 64 (3.14%) | **2** | 2 | 1▼ | 1▼ | 2 | 2 | Heterozygous *CFHR1/CFHR4* deletion |
| F | 4 (0.20%) | **2** | 2 | 3▲ | 3▲ | 2 | 2 | Heterozygous *CFHR1/CFHR4* duplication |
| G | 3 (0.15%) | **2** | 1▼ | 0▼ | 1▼ | 2 | 2 | Heterozygous *CFHR3/CFHR1* deletion  and heterozygous *CFHR1/CFHR4* deletion |
| H | 1 (0.05%) | **2** | 0▼ | 1▼ | 3▲ | 2 | 2 | Heterozygous *CFHR3/CFHR1* deletion and heterozygous *CFHR3* deletion and heterozygous *CFHR4* duplication |
| I | 15 (0.74%) | **2** | 1▼ | 2 | 3▲ | 2 | 2 | Heterozygous *CFHR3* deletion and heterozygous *CFHR4* duplication |
| J | 1768 (86.84%) | **2** | 2 | 2 | 2 | 2 | 2 | No copy number variation |

**Supplementary Table 1. Summary of copy number variations (CNVs) in the CFH/CFHR gene cluster detected in 2 036 general population.**

The number of cases and their percentages are shown for each CNV pattern. A solid-black downward-pointing triangle indicates small values (i.e., where the copy number of each gene is less than two) and a solid-black upward-pointing triangle indicates copy numbers of more than two. Although various CNV patterns were observed in the CFH/CFHR gene cluster, no CNV in the CFH gene was observed in any of the 2 036 cases.
