# Supplementary material for: CFH-CFHR1 hybrid genes in two cases of atypical hemolytic uremic syndrome
Source: J Hum Genet. 2023 Feb 9;68(6):427–30. doi: 10.1038/s10038-023-01129-1 (PMC10208952; doi:10.1038/s10038-023-01129-1)
Supplement: Supplementary file 6 — Supplementary Information [file 10038_2023_1129_MOESM6_ESM.docx]

**Supplementary Information**

Supplementary information is available at *Journal of Human Genetics*’ website.

**Supplementary Notes. Materials and methods. (.docx)**

**Supplementary Fig. 1. Discretized copy number of the *CFH/CFHR* gene cluster in 2 036 individuals in the general population.**

The heatmap shows the normalized sequence coverage of *CFH*, *CFHR3*, *CFHR1*, *CFHR4*, *CFHR2*, and *CFHR5* genes in 2 036 samples (Supplementary Notes). For the sake of clarity, samples with similar copy number changes are grouped vertically. Blue indicates low coverage, i.e., a low number of copies, and red indicates high coverage, i.e., a high number of gene copies. (.pdf)

**Supplementary Fig. 2. Copy number of the CFH/CFHR gene cluster in 2 036 individuals in the general population and two aHUS cases with hybrid genes.**

The heatmap shows the normalized sequence coverage of *CFH*, *CFHR3*, *CFHR1*, *CFHR4*, *CFHR2*, and *CFHR5* genes in 2 036 samples and two aHUS cases with hybrid genes, that are shown with “Patient-1” and “Patient-2” in the right side of the figure. Samples with similar copy number changes are grouped vertically. Blue indicates low coverage, i.e., a low number of copies, and red indicates high coverage, i.e., a high number of gene copies. (.pdf)

**Supplementary Table 1. Summary of copy number variations (CNVs) in the *CFH/CFHR* gene cluster detected in 2 036 general population.**

The number of cases and their percentages are shown for each CNV pattern. A solid-black downward-pointing triangle indicates small values (i.e., where the copy number of each gene is less than two) and a solid-black upward-pointing triangle indicates copy numbers of more than two. Although various CNV patterns were observed in the *CFH/CFHR* gene cluster, no CNV in the *CFH* gene was observed in any of the 2 036 cases. (.docx)
